# GPCR heteromers: An overview of their classification, function and physiological relevance

**DOI:** 10.3389/fendo.2022.931573

**Published:** 2022-08-30

**Authors:** Natasha C. Dale, Elizabeth K. M. Johnstone, Kevin D. G. Pfleger

**Affiliations:** ^1^ Molecular Endocrinology and Pharmacology, Harry Perkins Institute of Medical Research and Centre for Medical Research, The University of Western Australia, Nedlands, WA, Australia; ^2^ Australian Research Council Centre for Personalised Therapeutics Technologies, Perth, WA, Australia; ^3^ School of Biomedical Sciences, The University of Western Australia, Nedlands, WA, Australia; ^4^ Dimerix Limited, Nedlands, Australia

**Keywords:** G protein-coupled receptors (GPCRs), heteromer, dimer, biased signalling, β-arrestin, endocytosis

## Abstract

G protein-coupled receptors (GPCRs) are capable of interacting to form higher order structures such as homomers and heteromers. Heteromerisation in particular has implications for receptor function, with research showing receptors can attain unique expression, ligand binding, signalling and intracellular trafficking upon heteromerisation. As such, GPCR heteromers represent novel drug targets with extensive therapeutic potential. Changes to ligand affinity, efficacy and G protein coupling have all been described, with alterations to these pharmacological aspects now well accepted as common traits for heteromeric complexes. Changes in internalisation and trafficking kinetics, as well as β-arrestin interactions are also becoming more apparent, however, few studies to date have explicitly looked at the implications these factors have upon the signalling profile of a heteromer. Development of ligands to target GPCR heteromers both experimentally and therapeutically has been mostly concentrated on bivalent ligands due to difficulties in identifying and developing heteromer-specific ligands. Improving our understanding of the pharmacology and physiology of GPCR heteromers will enable further development of heteromer-specific ligands with potential to provide therapeutics with increased efficacy and decreased side effects.

## Introduction

G protein-coupled receptors (GPCRs) consist of seven transmembrane domains. Upon activation, most GPCRs couple to heterotrimeric G proteins to produce physiological effects. GPCRs also engage in an array of interactions with other macromolecular complexes that play important roles in determining GPCR function. The GPCR family has an extremely broad range of physiological functions, particularly in relation to the number of non-olfactory GPCR encoding genes (1). One explanation for this is that higher order GPCR interactions can alter receptor function, thereby enabling this diversity. Indeed, the ability for GPCRs to form oligomers is now widely accepted, with research demonstrating their ability to form multi-receptor complexes with identical (homomers) and different (heteromers) receptor units both *in vitro* and *in vivo.* Both GPCR homomers and heteromers may display different biochemical properties from their monomeric counterparts (2), with potential alterations to all facets of their pharmacology. For example, one of the first indicators that GPCRs formed oligomeric species came from observations of cooperativity in ligand binding data (3, 4), suggesting the existence of GPCR homomers (5–8) and heteromers (9–11). While both GPCR homomers and heteromers can attain unique pharmacology, heteromerisation has the potential for increased diversification and complexity of signaling, as a result of the interaction of different receptors which have different structures, ligand binding pockets, effectors etc. As a result, GPCR heteromerisation has been a prominent area of GPCR research, and is therefore also the focus of this review. It is important to be aware that in both experimental and physiological conditions, GPCRs likely exist in mixed populations of monomers, homomers and heteromers. As a result, identifying and differentiating the pharmacology of the various oligomeric GPCR species, and in particular differentiating monomers from homomers, can be a challenge, and most studies do not differentiate between monomers and homomers unless they are specifically being investigated. Consequently, unless otherwise specified, this review will refer to single-receptor results as monomeric conditions.

Some GPCRs heteromerise to form a minimal functional unit, such as the Class C γ-aminobutyric acid (GABA) heteromeric receptor consisting of GABA_B1_ and GABA_B2_ subunits. This has been shown *in vivo* (12), with the subunits unable to mediate signal transduction or traffic to the plasma membrane when existing as monomeric receptors (12–14). Following initial reports of Class C GPCR heteromerisation, a wide range of Class C GPCRs were found to form such heteromers *in vivo* (15–17). It is now widely accepted that many Class C GPCRs require heteromerisation to form a functional signalling unit (18, 19). This form of heteromerisation is termed a “heteromeric receptor” as it is necessary for formation of a minimal functional unit (20). This differs from “receptor heteromers”, which are comprised of two or more minimal functional units that form a macromolecular complex with biochemical properties that differ from their individual components (20). As receptor heteromers have different biochemical properties from their respective monomers, they are effectively novel drug targets with the potential to achieve greater specificity and selectively. However, despite this they remain relatively unexplored as targets for therapeutic interventions.

This review will discuss receptor heteromers, with a focus on Class A GPCR heteromers, heteromer signalling and the effects of ligands upon heteromer function. Examples of heteromers implicated in pathological conditions will also be discussed, as well as strategies currently used to target heteromers. While not explored here, it is worth mentioning that receptor heteromer formation, stability and the nature of allosteric interactions that occur between protomers are also areas of active research and are revealing further complexity within GPCR systems (21, 22).

## Heteromer classification and investigation

A receptor heteromer is defined as a **“**macromolecular complex composed of at least two (functional) receptor units with biochemical properties that are demonstrably different from those of its individual components**”** (20). This definition is accompanied by a set of standards a proposed receptor heteromer must meet in order to be regarded as a heteromer with physiological relevance. The standards for confirming the physiological relevance of a receptor heteromer and the experimental methods used to do so have been discussed at length in several previous reviews (20, 23, 24), and as such will not be discussed in detail here. However, three criteria are widely accepted to demonstrate that a heteromer observed in a transfected *in vitro* setting has physiological relevance to an *in vivo* setting. Firstly, demonstrating the receptor protomers have the capacity to be in close proximity *in vivo* is an important initial step to validate the heteromeric complex. Appropriate evidence for this includes confirming the receptor protomers are found within the same cell and are sequestered within the same cellular compartments in either native or primary cells (20, 23, 24). This is most commonly achieved through immunoelectron microscopy, coimmunoprecipitation and proximity-based biophysical techniques such as Fluorescence Resonance Energy Transfer (FRET) (23) and proximity ligation assays (25–27). These techniques can also be used to give evidence for heteromer formation directly, through demonstrating the distance-dependent association of receptor protomers within relevant physiological environments.

Secondly, the heteromer should demonstrate biochemical properties distinct from its respective monomers (20, 23, 24). Different biochemical properties can arise as a result of allosteric modulation between protomers (24) resulting in unique signalling, trafficking, ligand binding or other pharmacological properties (23). This criterion can also be fulfilled through the confirmation of a heteromer-specific ligand (24). Common methods employed to observe the biochemical properties of heteromers include ligand-binding assays to investigate changes to ligand affinity, assays to observe modulation of signal transduction cascades by heteromers and assays that investigate the trafficking of heteromers, especially in regards to their trafficking to the cell surface and agonist-mediated internalisation (23).

**Figure 1:**
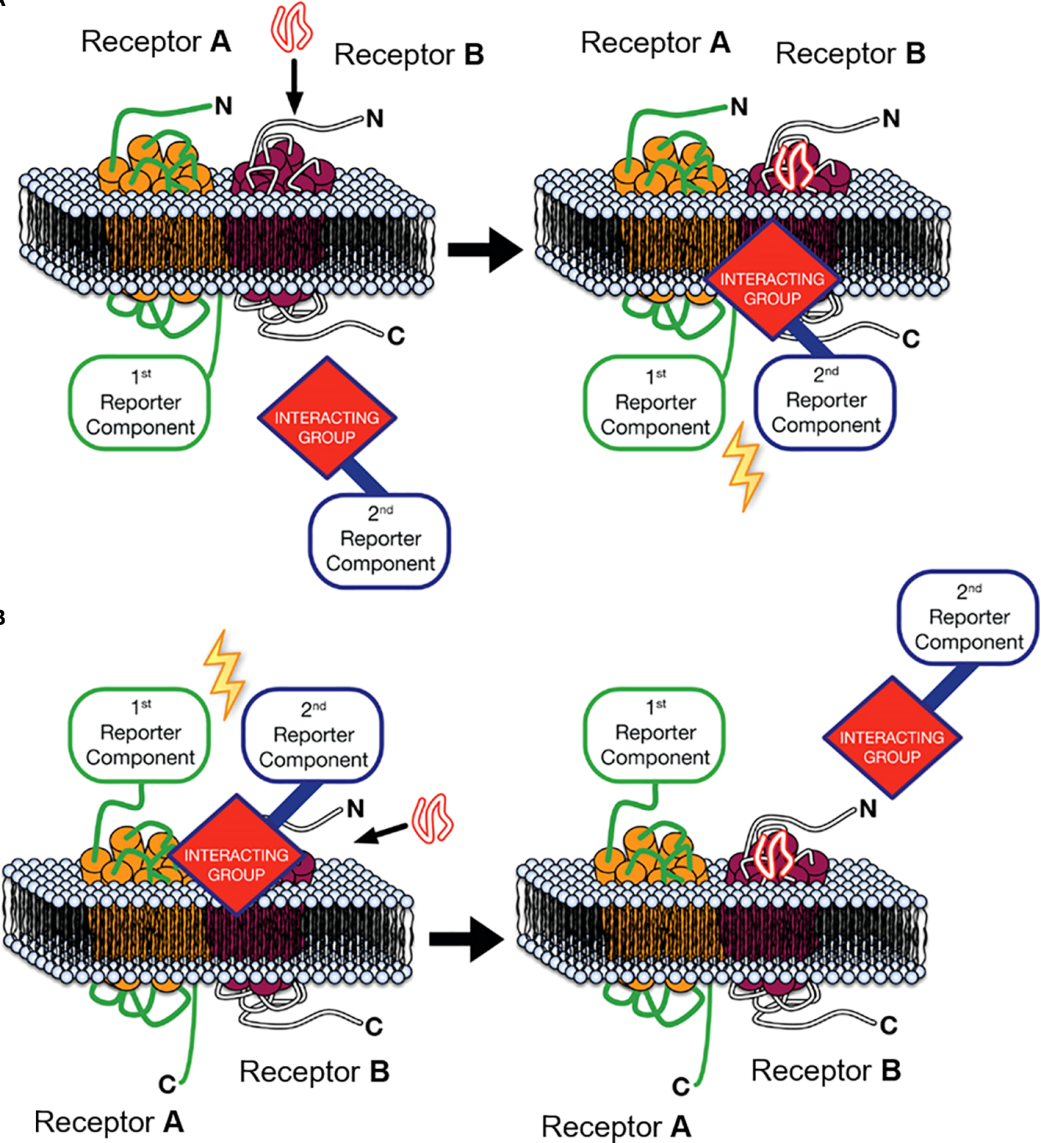
Receptor-Heteromer Investigation Technology (Receptor-HIT). Receptor A is fused to the first reporter component, Receptor B is unlabelled with respect to the reporter system, and a receptor-interacting group is linked to the complementary second reporter component. **(A)** The first reporter component is fused to the C-terminus of Receptor A and an intracellular interacting group is recruited to the heteromer upon stimulation with an agonist selective for Receptor B, **(B)** or ligand binding to the heteromer is assessed by fusing the first reporter component to the N-terminus of Receptor A and using a fluorescently-labelled ligand specific to receptor B as the interacting group/second reporter component combination. Modified from Jaeger et al. (31) under a Creative Commons Attribution License (CC BY). Full terms provided at https://creativecommons.org/licenses/by/3.0/.

One approach that can provide key experimental evidence to address this second criterion is Receptor-Heteromer Investigation Technology (HIT). This technology utilises a proximity-based biophysical technique, most commonly Bioluminescence Resonance Energy Transfer (BRET), to monitor receptor-receptor proximity in a ligand-dependent manner (28–30). The Receptor-HIT configuration, shown in **Figure 1A**, consists of a receptor tagged with a proximity-reporter component, an untagged receptor and an interacting protein tagged with the complementary proximity-reporter component. Upon addition of a ligand selective for the untagged receptor, the interacting protein will be recruited to the heteromer. This interacting protein, such as β-arrestin, is usually recruited specifically to the untagged receptor as part of prototypical signalling events following agonist-induced recruitment. However, on occasion, it may be recruited to the tagged receptor when it is in a heteromer complex with the untagged receptor, ie. as a result of transactivation (32). In addition to reporting receptor proximity, the Receptor-HIT assay also generates functional information as a result of the recruitment of the interacting protein, and therefore the assay can also reveal novel biochemical properties of the heteromer (28). Indeed, unique β-arrestin recruitment, G protein recruitment and other pharmacological properties have been observed using Receptor-HIT for multiple heteromeric complexes (29, 32–42). Receptor-HIT can also be configured to utilise a ligand labelled with the complementary proximity-reporter component, rather than an interacting protein as shown in **Figure 1B**. When used with the NanoBRET ligand-binding assay (43), heteromerisation can be monitored through fluorescent ligand binding to an untagged receptor that is adjacent to an N-terminally Nluc tagged receptor. Receptor-HIT is a powerful tool that can be used to validate receptor heteromers by simultaneously demonstrating heteromer formation and unique heteromer function, both of which are vital pieces of evidence to ascertain the potential physiological relevance of a receptor heteromer observed *in vitro*.

The last criterion required to demonstrate physiological relevance of a heteromer is that disruption of heteromer function should be observable upon the loss of a functional receptor heteromer (23, 24). Methods to disrupt heteromer formation include the use of membrane-permeable peptides targeted to the site of interaction between heteromer subunits, transgenic animals that express protomers incapable of forming heteromers (23), as well as knockout animal models that do not express one of the appropriate protomers (24). Once a model of heteromer disruption has been produced, there are many techniques that can be employed to demonstrate disruption to heteromer assembly and function. This includes comparison of functional characteristics between systems that express the relevant receptor heteromers and those that do not, utilising heteromer-specific antibodies that are able to detect heteromers (23, 44), as well as utilising bivalent and heteromer-selective ligands to show disruption to heteromer assembly and function (23).

For a receptor heteromer to be accepted as a heteromer with physiological relevance it must meet at least two of the three criteria outlined above in accordance with NC-IUPHAR guidelines (24). Historically, demonstrating these properties of receptor heteromers has been difficult due to a lack of appropriate experimental tools to investigate heteromer properties. With the use of the technologies outlined above, the ability to verify the existence of receptor heteromers in native tissue has become more achievable. This has led to an increase in the amount of receptor heteromers being identified and pharmacologically characterised.

## Effects of Heteromerisation

Critical features of receptor heteromers are their unique biochemical properties, which are distinct from the respective protomers (20, 23, 24). These unique biochemical properties can relate to any aspect of the heteromer’s pharmacology, including ligand binding, signalling and trafficking. Allosteric interactions between the complementary monomers within the heteromer are the most commonly suggested mechanism for heteromer-specific signalling (20, 45, 46).

### Ligand binding

In receptor heteromers, allosteric modulation by the complementary monomer resulting in different ligand binding properties is a commonly observed novel biochemical property (47). The effect and extent of these allosteric interactions is highly variable. Positive allosteric modulation has been observed at the δ-μ opioid receptor heteromer, with an increase in maximum binding (Bmax) of the µ opioid receptor ligands DAMGO and morphine observed in the presence of δ opioid receptor ligands (48). Positive allosteric modulation also appears to occur within the CCR2b-CCR5 chemokine receptor heteromer (9). Here, in competition binding assays, CCR5 ligands were only able to compete for CCR2b radioligand binding when CCR2b and CCR5 were coexpressed. Similarly, in cells coexpressing CCR2b and CCR5 and treated with CCR2b radioligand, there was a left shift in the CCR2b ligand displacement curve. Negative allosteric modulation has been observed in our studies using the Receptor-HIT assay (Johnstone et al. (43)). We observed a decrease in the affinity of fluorescently-labelled angiotensin II and propranolol when bound to the respective receptor heteromerised within the AT_1_-β_2_ heteromer, compared to the affinity of each ligand at the respective non-heteromerised receptor.

### G protein coupling

Receptor heteromers are also capable of modulating G protein coupling, as seen with the α_2A_ adrenergic-μ opioid receptor heteromer (49) in which μ opioid receptor activation with morphine decreased the efficacy of noradrenaline in signaling through G_i_ due to resulting conformational changes in the α_2A_ receptor. Additionally, increased potency of the μ agonist-δ antagonist eluxadoline is observed in the presence of δ-μ heteromers compared to the potency in the presence of μ opioid receptors only (50), however, whether this is due to occupancy of both targets or heteromer-specific binding properties of eluxadoline is unclear. Changes in the magnitude of signalling in response to agonist-induced activation is a commonly observed functional change as a result of heteromerisation, with recent reports indicating roles for heteromer-specific signalling mechanisms in the pathophysiology of nervous system disorders such as Huntington’s disease (51) and Parkinson’s disease (52).

Heteromerisation is also capable of changing the predominant second messenger through which a protomer signals. Examples of this can be seen with the switch from Gα_i_ coupling to Gα_s_ coupling of the CB_1_ cannabinoid receptor when heteromerised with the D_2_ dopamine receptor (53), and a switch from Gα_s_ to Gα_i_ coupling of the D_1_ dopamine receptor when heteromerised with the H_3_ histamine receptor (54). When δ and μ opioid receptors heteromerise, they switch G protein coupling preference from predominantly Gα_i_ to Gα_z_ (55). Additionally, melatonin is able to activate both the MT_2_ melatonin receptor-mediated Gα_i_ cascade as well as the 5-HT_2C_ serotonin receptor-mediated Gα_q_ cascade through transactivation within the MT2-5-HT_2C_ receptor heteromer (56). Recently, we have discovered novel G protein coupling by the heteromer that forms between the angiotensin II AT_2_ receptor and the bradykinin B_2_ receptor. Although neither receptor recruits Gα_z_ following treatment with agonist, upon their coexpression Gα_z_ is recruited to the heteromer in a bradykinin dependent manner (57).

### Trafficking profiles

Research into receptor heteromer trafficking has been largely focused on synthesis and trafficking to the plasma membrane, with the goal to determine whether heteromerisation occurs before or after reaching the plasma membrane. Results from these studies have been mixed and suggest that the site of formation of receptor heteromers is dependent upon the specific receptors involved.

Much of the early research on the location of GPCR oligomer formation investigated homomers (58–62). This research revealed GPCR oligomers were capable of forming constitutively on the plasma membrane, with further studies also demonstrating that some class A homomers formed prior to trafficking to the cell surface (63–65). It is, however, apparent that homomers and heteromers differ from each other in the impact oligomerisation has upon their function (66). As such, heteromer-based studies were also needed to elucidate the distinct trafficking profiles of heteromers.

Early reports on trafficking of the δ-μ opioid receptor heteromer suggested the heteromeric complex forms at the plasma membrane in response to G protein recruitment (67). However, subsequent findings disagreed with this, instead giving evidence for the heteromer forming constitutively in the endoplasmic reticulum (ER) and Golgi apparatus prior to trafficking to the plasma membrane as a heteromer (55). Hasbi et al. (55) attempted to reconcile the differences in observations by suggesting Law et al. (67) staggered transfection of the two receptors into cell culture, as they found this to produce non-interacting receptor populations. Further evidence within the literature also supports the finding that δ-μ opioid receptor heteromers form prior to trafficking to the cell surface. Décaillot et al. (68) reported heteromer formation in the ER and storage within the Golgi apparatus prior to trafficking to the cell surface mediated by receptor transport protein 4. This pathway of ER synthesis and subsequent plasma membrane trafficking is supported by data from other class A GPCR heteromers, which have also been found to form prior to trafficking to the cell surface (69–71). These data, combined with evidence indicating some Class A GPCRs are not expressed on the plasma membrane when transfected alone into heterologous cells, has led to the suggestion that heteromerisation may play a functional role in the maturation and targeting of Class A GPCRs to the plasma membrane (72). This is supported by studies that show co-expression of mutated GPCRs with wild-type GPCRs results in the sequestration of a mutant-wild-type heteromeric complex within the ER, preventing expression of the heteromeric complex on the plasma membrane (73–77). It should be noted that these studies utilise a mutant of the wild-type receptor used. While this gives a clear demonstration of the effect of mutation on homomer formation and resulting subcellular sequestration, the extent to which this can be applied to heteromers in unclear. Nonetheless, studies of the α_1D_ adrenoceptor demonstrated that heteromerisation with α_1B_ or β_2_ adrenoceptors is required for α_1D_ adrenoceptor targeting to the plasma membrane (69, 71), with similar results also shown for targeting of olfactory receptors to the plasma membrane (70). From this, it is apparent heteromerisation plays a role in the targeting of some Class A GPCRs to the plasma membrane, however the prevalence of this property among heteromers is yet to be elucidated. Further heteromer studies utilising mutated and wild-type receptors will assist in developing our understanding of this facet of heteromer function.

Similar to heteromer cell surface trafficking, internalisation pathways also appear to vary widely depending on the specific heteromer. δ-κ opioid receptor heteromers do not undergo substantial internalisation upon stimulation with the non-selective opioid agonist etorphine, resulting in a change in internalisation kinetics of δ opioid receptors that show high levels of internalisation as monomers and homomers (78). This trend of one protomer exerting a dominant effect in relation to internalisation trafficking has also been observed with other GPCR heteromers (10, 79, 80). An example of this is provided by Terrillon et al., (81) where they demonstrate the V_2_ vasopressin receptor has a dominant effect over the V_1A_ vasopressin receptor when stimulated with a non-selective agonist, causing the heteromer to undergo prolonged trafficking with localisation to endosomes. This profile is consistent with observations of trafficking of V_2_ monomers, but significantly changes the trafficking profile of V_1A_, which undergoes rapid recycling as a monomer (81–83). Subsequently, Terrillon et al., (81) demonstrated that upon activation of V_1A_ with a selective agonist, the heteromer switched to exhibit a V_1A_ dominant trafficking profile. These data suggest that the V_1A_-V_2_ heteromer trafficking profile is dependent upon the activated protomer. If the activation-dependent switching of trafficking profiles is found to be a common feature of receptor heteromers this adds another dimension of complexity to GPCR function and signalling that must be taken into account in the development of both non-heteromer targeted and heteromer targeted therapeutics.

### Effect of altered trafficking on signalling

The diversity in changes to trafficking profiles resulting from heteromerisation has implications not only for heteromer formation, cell localisation and cell surface expression, but also for signalling.

An example where this relationship is particularly apparent is the PAR1-PAR2 proteinase-activated receptor heteromer. The PAR1-PAR2 heteromer internalises as a unit both constitutively and upon cleavage of PAR1**’**s N-terminus by thrombin. The cleaved N-terminus sequence acts as a tethered ligand for PAR1 that is also capable of transactivating PAR2 (84, 85). Following stimulation, the heteromer co-internalises to endosomes with β-arrestin leading to sustained β-arrestin-mediated ERK1/2 activation in the cytoplasm (86). This profile is unique to the heteromer, as the observed trafficking aligns with the PAR1 protomer, while PAR2 trafficking switches from stable cell surface expression to undergoing constitutive internalisation along with PAR1 in addition to thrombin-stimulated internalisation (86–88). Additionally, despite acquiring the trafficking profile of PAR1, β-arrestin recruitment follows the pattern seen with the PAR2 protomer upon activation by PAR2 agonist SLIGKV, with stable β-arrestin interactions formed that co-internalise to endosomes resulting in prolonged cytosolic ERK1/2 activation (89, 90), as opposed to transient β-arrestin interactions observed with PAR1 protomers (91). The unique β-arrestin interaction and internalisation properties of the PAR1-PAR2 heteromer appear to have functional consequences for the signalling of the heteromer-β-arrestin complex, as decreased PAR1-mediated ERK1/2 nuclear translocation was observed with activation of the heteromer compared to PAR1 monomers. Lin and Trejo (86) suggest this change in signalling arises from the PAR2 protomer causing prolonged cytoplasmic ERK1/2 activation while simultaneously limiting PAR1 mediated ERK1/2 translocation to the nucleus. These changes to endosomal β-arrestin-mediated signalling may be important to the PAR1-PAR2 heteromer**’**s apparent pathological roles (92, 93), however research to elucidate this further is yet to be conducted.

To date, few studies have characterised the effect heteromerisation-induced altered trafficking has upon physiological function. However, the accumulating body of evidence showing changes to trafficking upon heteromerisation offers exciting potential for therapeutically targeting this aspect of heteromer pharmacology.

### β-arrestin complexes

GPCR desensitisation and internalisation is largely mediated by β-arrestins (94). It is now well established that β-arrestins also mediate secondary signalling complexes as discussed briefly above (95–97). Given the changes to internalisation and trafficking observed with heteromerisation, it is not surprising that changes in receptor interactions with β-arrestin are also observed.

The AT_1_-AT_2_ angiotensin receptor heteromer associates with β-arrestin2 following stimulation but does not subsequently internalise (33). This is a change in β-arrestin interactions for both receptors, as activated AT_1_ recruits β-arrestin followed by internalisation of the receptor (98, 99), while AT_2_ does not associate with β-arrestin following activation and consequently does not undergo internalisation (99). Porrello et al. (33) suggested this change may be a consequence of AT_2_ cross-inhibiting the internalisation of AT_1_ and that this interaction may play a role in the antagonistic influence of AT_2_ on the function of AT_1_ that is also observed (100). Similarly, heteromerisation of the α_1A_-adrenergic and CXCR2 receptors results in stable recruitment of β-arrestin2 in response to noradrenaline (32), in contrast to α_1A_ adrenoceptor monomers, which interact weakly with β-arrestins upon stimulation with noradrenaline (101). More recently, it has been shown that heteromerisation of AT_1_ and β_2_AR results in increased β-arrestin recruitment to β_2_AR upon co-stimulation of both receptors (37). These examples show changes to β-arrestin interactions as a result of receptor cross-talk following heteromerisation. Subsequent changes to downstream signalling properties are yet to be elucidated for the given examples, however the changes outlined could potentially have significant effects on downstream function.

Heteromerisation is also capable of changing signalling from G-protein dominant to β-arrestin dominant. CXCR4-CXCR7 chemokine receptor heteromer formation results in a decrease in the Gα_i_-dependent signalling of CXCR4 and leads to an increase in β-arrestin mediated activation of ERK1/2, p38 MAPK and stress-activated protein kinase (SAPK) cascades (102). δ-μ opioid receptor heteromerisation and subsequent activation results in the recruitment of β-arrestin2, which leads to a profile of sustained β-arrestin mediated ERK1/2 phosphorylation, in contrast to the transient ERK1/2 phosphorylation exhibited by μ receptor homomers (103). Change in the relative signalling of G protein mediated and β-arrestin mediated pathways following activation holds implications for receptor function, similar to the switching of G protein coupling previously mentioned. Despite this, changes to heteromerisation-induced β-arrestin-mediated signalling have not been thoroughly investigated. Future research may shed further light on the effects of heteromerisation on secondary signalling pathways, and how these changes may be exploited in the future for pharmacological intervention.

## Heteromer-targeted ligands

Two approaches have been used to produce ligands that target heteromers. Heteromer-specific ligands and bivalent ligands are both designed to selectively activate or inhibit heteromers over their constitutive monomers (104). Both types of ligands have been explored for research and therapeutic purposes.

### Heteromer-selective ligands

Heteromer-selective ligands are novel ligands that are developed to have selectivity for a specific heteromeric complex. The ultimate aim in development of these ligands is to establish heteromers as standalone targets for novel drug action without undue modulation of the monomeric receptors that constitute the heteromer.

δ opioid receptor antagonist and κ opioid receptor agonist 6’-guanidinonaltrindole (6’-GNTI) appears to act as a heteromer-selective ligand at the δ-κ heteromer (105). The signalling profile elicited by 6’-GNTI in the presence of both δ and κ opioid receptors could not be attributed to the synergistic effect of δ receptor antagonism with κ receptor agonism, with Ca^2+^ release stimulated by 6’-GNTI in the presence of δ and κ opioid receptors over five times more potent than in cells expressing only the κ opioid receptor, suggesting activation of heteromer-unique signalling. Also consistent with heteromer-specificity, 6’-GNTI exhibited high affinity binding in δ and κ opioid receptor expressing cells, while the opioid ligands norbinaltorphimine and naltrindole exhibited affinity values that were not consistent with the affinities observed in cells expressing either receptor alone, giving evidence for a heteromer-specific binding complex in these cells.

Waldhoer et al. (105) report 6’-GNTI induced analgesia in response to intrathecal injection, but not intracerebroventricular injection in mice. Given that there is evidence for δ-κ heteromer formation within the spinal cord (106), but evidence against δ-κ heteromerisation within the brain (107), 6’-GNTI’s efficacy in the spinal cord but not the brain gives further evidence for its action as a heteromer-selective ligand. Finally, 6’-GNTI-induced analgesia was blocked by δ opioid receptor antagonist naltrindole and a δ-κ heteromer-selective bivalent antagonist KDN-21, with the heteromer-selective antagonist blocking analgesia with increased potency over the single receptor antagonist. Taken together, these data strongly support 6’-GNTI acting as a heteromer-biased ligand to produce analgesia in the spinal cord (105). These studies were the first strong evidence describing a heteromer-selective ligand, highlighting the potential for the therapeutic targeting of heteromers. Indeed, 6’-GNTI was recently shown to produce prolonged anti-nociception compared to single receptor δ or κ receptor agonists in rats (108), giving further support for 6’-GNTI and specific targeting of the δ-κ heteromer as a promising avenue for the development of novel pain treatments.

However, despite these promising results, other studies investigating heteromer-specific ligands have given mixed results.

Rashid et al. (11) investigated the effect of the purported D_1_ agonist SKF83959 on the signalling of the D_1_-D_2_ dopamine receptor heteromer. Upon application of SKF83959, the D_1_-D_2_ heteromer signalled through Gα_q_ as opposed to Gα_s_ and Gα_i_ for the respective monomers. As such, Rashid etal. (11) suggested SKF83959 elicits heteromer-selective activity through binding to D_1_ as well as D_2_ when it is in the heteromeric conformation with D_1_. However, these findings were challenged by Lee et al. (109) who were unable to produce significant phospholipase C (PLC) activation in HEK-293 cells coexpressing D_1_ and D_2_ receptors. At high concentrations of SKF83959, a small increase in PLC activation was observed, however this was shown to also occur in wild-type HEK-293 cells and cells expressing only D_1_ receptors. From these results Lee et al. (109) suggest that SKF83959 does not act as a heteromer-selective ligand that activates Gα_q_ through the D_1_-D_2_ heteromer, but is instead a typical D_1_ receptor partial agonist. They suggest that the previously reported PLC activation is attributable to the ligand’s effects on off-target receptors due to the high concentrations of ligand often used in SKF83959 studies (11, 110). These conflicting results highlight the difficulty in identifying true heteromer-biased ligands and the importance of tightly controlled experimental designs to ensure the validity of any experiments investigating such ligands.

Gomes et al. (111) gives an example of the potential clinical relevance of heteromer-targeted ligands with the proposed δ-μ opioid receptor heteromer-specific ligand CYM51010. CYM51010 showed activity only in cells expressing both δ and μ receptors. This activity was blocked by a heteromer-specific antibody. CYM51010 exhibited higher EC_50_ values for β-arrestin recruitment at δ and μ receptors compared to the heteromer, while exhibiting higher EC_50_ values for G protein recruitment at the heteromer compared to the respective monomers. Given this evidence of specific G protein biased activity at the δ-μ heteromer, the subsequent finding that CYM51010 produces antinociception with decreased tolerance compared to morphine provides an important proof-of-principle for the utility of heteromer-specific ligands as potential therapeutics. Further research also provided two additional δ-μ heteromer-specific ligands that show potential as novel antinociceptive therapeutics (112, 113).

Despite the experimental and therapeutic potential of heteromer-specific ligands, their development and discovery remain limited. This is due to the difficulty in identifying compounds that display the desired qualities without extensive screening of compound libraries (111). Difficulties in the identification and development of heteromer-specific ligands have slowed the progression of research utilising such compounds. As a result, techniques using bivalent ligands have been developed to attempt to overcome these practical limitations.

### Bivalent ligands

Bivalent ligands consist of two pharmacophores joined by a linker, allowing dual binding to each receptor within a protomer. Bivalent ligands can be developed for homomers, whereby the pharmacophores may be identical or different. Many such ligands have been developed for research as well as potential therapeutic purposes (114–117). Bivalent ligands can also be developed for heteromers, whereby each ligand is targeted to one constituent receptor within the heteromer. They have been developed to study and screen receptor heteromers for their specific effects on signalling (118). Due to their utilisation of existing ligands with known properties, the development of bivalent ligands is more readily achievable in comparison to novel heteromer-selective ligands.

An example strategy of bivalent ligand development is described by Harvey et al. (119), who combined low affinity δ opioid receptor ligands with high affinity μ opioid receptor ligands. The resulting bivalent ligands display increased affinity for the δ-μ heteromer with decreased affinity for δ receptor homomers. This method provides an alternative strategy of producing ligands with heteromer-specific properties that overcome some of the practical challenges of screening for new novel compounds. However, the utility of bivalent ligands is limited by the properties of the constituent ligands, and as such do not erase the need for research into novel heteromer-biased and heteromer-selective ligands.

An early study utilising bivalent ligands demonstrated their usefulness in heteromer identification and verification. Daniels et al. (120) varied the length of the linker within a bivalent ligand comprised of a μ opioid receptor agonist and δ opioid receptor antagonist. Bivalent ligands with suboptimal linker length were associated with the development of tolerance, while ligands with optimal length linkers to bind to δ-μ heteromers were associated with no significant tolerance developing. Given that previous studies had established δ opioid receptor blockade abolished tolerance in response to chronic administration of a μ opioid receptor agonist (121, 122), Daniels et al. (120) offers evidence for the existence of δ-μ heteromers *in vivo* through the use of bivalent ligands. Additionally, this study also showcases the potential for using bivalent ligands to specifically target receptor heteromers and their unique pharmacology to provide improved therapeutic outcomes.

The majority of early studies investigating bivalent ligands were limited by their inability to demonstrate a large difference in the affinity of bivalent ligands compared to monovalent ligands (120, 123). This is an important factor in demonstrating binding to a receptor heteromer, as a large difference in affinity can be expected when comparing monomers to heteromers due to the additive affinity values of two binding sites (124). This caveat was overcome through the use of high-resolution crystal structures to map GPCR-ligand complexes and the utilisation of this information to guide the development of heteromer-targeted bivalent ligands (124). Hübner et al. (124) successfully created a D_2_-NTS_1_ dopamine-neurotensin receptor heteromer-biased bivalent ligand that demonstrated affinity three orders of magnitude higher than that seen for D_2_ receptors alone. Subsequently, they also observed a switch in signalling properties of the bivalent ligand from acting as a D_2_ agonist in cells expressing only the D_2_ receptor, to attenuating D_2_ signalling when the D_2_-NTS_1_ heteromer was present. This demonstrated that appropriately designed bivalent ligands have the ability to elicit receptor activation profiles unique to the targeted receptor heteromer. The success of this method in improving bivalent ligand specificity has exciting implications for their future use in both the investigation of receptor heteromers as well as clinical applications.

Other recent examples include the application of bivalent ligands to μ-D_4_ heteromers, implicated in chronic pain and addiction processes (125) and the μ-CCR5 receptor heteromer as a potential therapeutic to prevent neurological complications in HIV (126). These studies show an increased awareness for the importance of demonstrating high affinity values for heteromer-targeted bivalent ligands, as do recent reports on homomer-targeted bivalent ligands (127). The importance of this when developing heteromer-targeted bivalent ligands is exemplified by Peterson et al. (128), who, despite providing evidence for a μ-mGluR5 receptor heteromer bivalent ligand efficacious in reducing neuropathic pain, suggest the ligand is not specifically targeting heteromers. This is due to their observation that an analog of the bivalent ligand with a linker too short to span the binding sites of both receptors when heteromerised showed similar efficacy to the bivalent ligand with an appropriate linker length to target the heteromer. As such, it is more likely that the efficacy of the bivalent ligand is a result of synergistic effects of the bivalent ligand acting at each receptor as monomers. However, the opposite has been found in a previous study on the same heteromer, in which the bivalent ligand with optimal linker length exhibited higher potency than the suboptimal linker-length bivalent ligand (129). These studies investigated the effect of the bivalent ligand in differing models of nociception; neuropathic hyperalgesia (128) and bone cancer-induced chronic hyperalgesia (129), as such differing signal transduction mechanisms between the two models may play a role in the differing results observed. However, further research is required to determine the effect of this and other potential influential factors.

## Examples of physiological relevance to disease states

### 5-HT_2A_-mGluR2 Heteromers in Schizophrenia

The 5-HT_2A_ serotonin receptor and the mGluR2 glutamate receptor signal through Gα_q_ and Gα_i_ respectively, and are both targets of atypical antipsychotics. Antipsychotics used to target 5-HT_2A_ act as inverse agonists at the receptor to decrease Gα_q_ signalling. Antipsychotics targeting mGluR2 have an agonistic affect increasing signalling through Gα_i_ (130). It has been demonstrated that 5-HT_2A_ and mGluR2 form a heteromer in native tissue (131) and that in a non-pathological state this heteromer acts to modulate signal transduction through each receptor protomer to increase Gα_i_ signalling and decrease Gα_q_ signalling (130). Untreated patients with schizophrenia have been observed to exhibit upregulation of 5-HT_2A_ alongside downregulation of mGluR2 (131), and this is postulated to lead to a sub-optimal ratio of receptors for heteromerisation, leading to decreased heteromer formation in schizophrenia (130, 132). From these findings it has been suggested that combination therapy of a 5-HT_2A_ inverse agonist such as risperidone (133) and a mGluR2 agonist such as LY379268 will mimic the signalling balance caused by the presence of the receptor heteromer, leading to improved patient outcomes with preclinical experiments supporting this hypothesis (130). Despite this seemingly straightforward interaction between 5-HT_2A_ and mGluR2 mediated through heteromerisation, further research has revealed complexities in the function of this heteromer that may inform future targeting strategies (134, 135).

### A_2A_-D_2_ Heteromer in the basal ganglia

GABAergic striato-pallidal neurons are involved in processing of locomotor activity along with other functions of the basal ganglia such as reward mechanisms (136). A_2A_ adenosine receptors and D_2_ dopamine receptors are both highly expressed within these neurons and have been demonstrated to form heteromers in native tissue (27, 137, 138). It has been established that cross-talk occurs between subunits within the heteromer, with A_2A_ agonists decreasing the efficacy of D_2_ agonists (139). In addition to these unique pharmacological characteristics of the heteromer, which have implications for adenosine and dopaminergic signalling within the basal ganglia, recent findings by Taura et al. (140) demonstrate that maintenance of normal prepulse inhibition and haloperidol-induced catalepsy are both dependent on signalling from the A_2A_ receptor, giving behavioural evidence for interaction between A_2A_ and D_2_ pathways. These data establish A_2A_ and the A_2A_-D_2_ heteromer as potential novel drug targets in the treatment of dopaminergic system pathologies such as Parkinson’s Disease (141). This has already progressed to clinical applications, with A_2A_ antagonist istradefylline approved as a treatment for Parkinson’s Disease in Japan (142).

### AT_1_-CB_1_ Heteromers in liver fibrosis

Upregulation of CB_1_ cannabinoid receptors occurs in active hepatic stellate cells and contributes to liver fibrosis (143). Treatment of CB_1_ with an antagonist results in decreased liver fibrosis (143) and has also been found to improve liver steatosis (144). The angiotensin II type 1 receptor (AT_1_) is also expressed within activated hepatic stellate cells and plays a profibrotic role, with AT_1_ antagonists also decreasing hepatic fibrosis (145, 146). Historically, therapeutic targeting of cannabinoid receptors has been complicated due to the prevalence of central nervous system (CNS) side effects (147–149). Given this, the proposal of an upregulated AT_1_-CB_1_ receptor heteromer within activated hepatic stellate cells during liver fibrosis (150) presents a potential novel target in the treatment of liver fibrosis, and this has formed the basis of a recent patent for AT_1_-CB_1_ heteromer biased antibodies for the treatment of liver fibrosis (151). This potential treatment aims to provide a strategy to combat liver fibrosis, which is not susceptible to the adverse CNS effects of existing CB_1_ antagonists. By producing a heteromer-biased therapeutic, increased efficacy at hepatic stellate cells leading to decreased fibrosis should be achievable, alongside decreased CNS side effects through diminished activity of the therapeutic at CB_1_ monomers and homomers within the CNS and other non-target peripheral tissues.

## Conclusion

Changes to GPCR function as a result of heteromerisation presents a complex and potentially highly influential facet of GPCR signalling. In order to properly understand, and appropriately target GPCRs, the impact of heteromerisation must be elucidated. The development of new technologies that can monitor heteromer formation and pharmacology under increasingly physiologically relevant conditions will be important to aiding advances in heteromer identification. This will in-turn facilitate the development of heteromer-targeted therapies into mainstream application, offering novel therapeutic opportunities with the potential to improve treatment outcomes.

## Author contributions

All authors listed have made a substantial, direct, and intellectual contribution to the work and approved it for publication.

## Funding

This work was supported by an Australian Government Research Training Program Scholarship and a University of Western Australia Baillieu Research Scholarship (ND), as well as an Australian Research Council Industrial Transformation Training Centre IC170100016 Postdoctoral Fellowship (EJ).

## Conflict of interest

KP is Chief Scientific Advisor to Dimerix, of which he has a shareholding.

The remaining authors declare that the research was conducted in the absence of any commercial or financial relationships that could be construed as a potential conflict of interest.

## Publisher’s note

All claims expressed in this article are solely those of the authors and do not necessarily represent those of their affiliated organizations, or those of the publisher, the editors and the reviewers. Any product that may be evaluated in this article, or claim that may be made by its manufacturer, is not guaranteed or endorsed by the publisher.
